# Supplementary material for: Mechanism of microtubule stabilization by taccalonolide AJ
Source: Nat Commun. 2017 Jun 6;8:15787. doi: 10.1038/ncomms15787 (PMC5467209; doi:10.1038/ncomms15787)
Supplement: Supplementary Information — Supplementary figures. [file ncomms15787-s1.pdf]

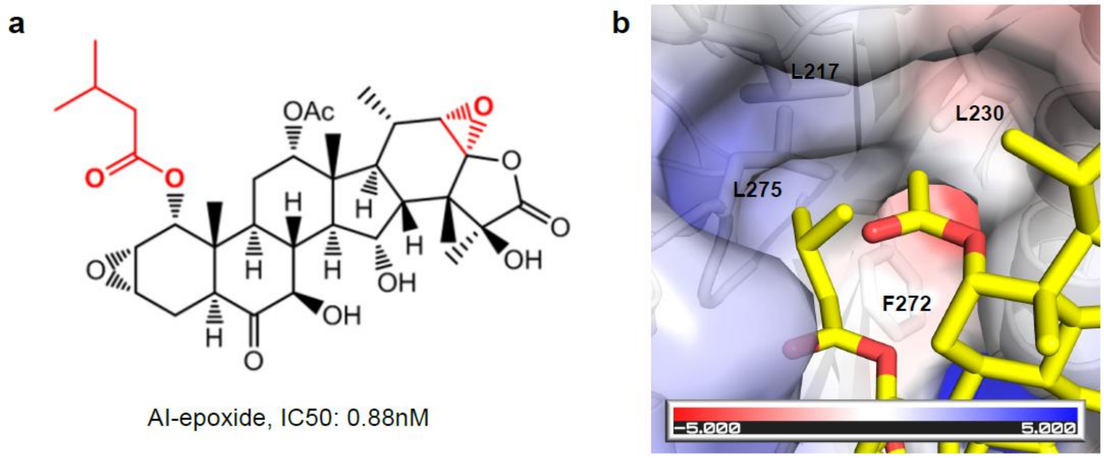

Supplementary Figure 1. **Docking model of tubulin-AI-epoxide.** (a) Chemical formula of AI-epoxide. Its epoxide and isovaleryloxy group are colored red. (b) The isovaleryloxy group of AI-epoxide sticks to a hydrophobic pocket. Electrostatic surface potential of the β-tubulin is shown. Blue and red (+/-5 kT/e) indicate the positively and negatively charged areas, respectively, of the protein. AI-epoxide (yellow) and the hydrophobic residues (grey) forming the pocket are shown in sticks.

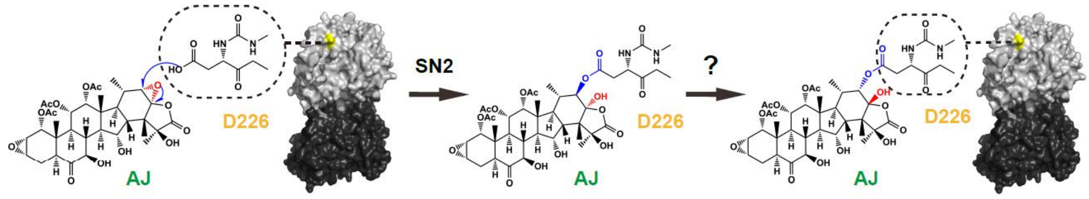

Supplementary Figure 2. **The covalent bond between AJ and βD226 raises a question of chirality of the epoxide group.** SN2 reaction: the carboxyl group of βD226 attacks the C22 as a nucleophile, resulting in the epoxide ring opening and an ester bond between βD226 and AJ. However, the chiralities of the resulting ester bond (blue) and the hydroxyl group (red) by a SN2 reaction are not consistent with what we observed in the crystal structure.

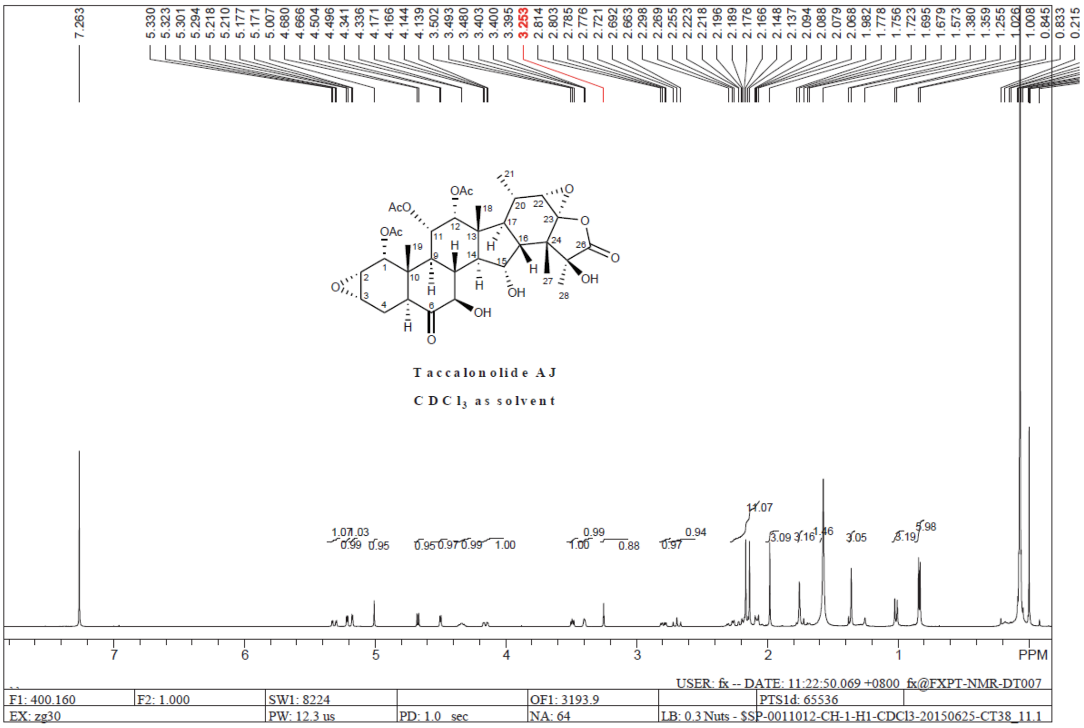

Supplementary Figure 3. **^{1}H NMR for taccalonolide AJ.** Components in $CDCl_3$, at room temperature (ppm Downfield from TMS). The chemical shift of H22 is colored red.

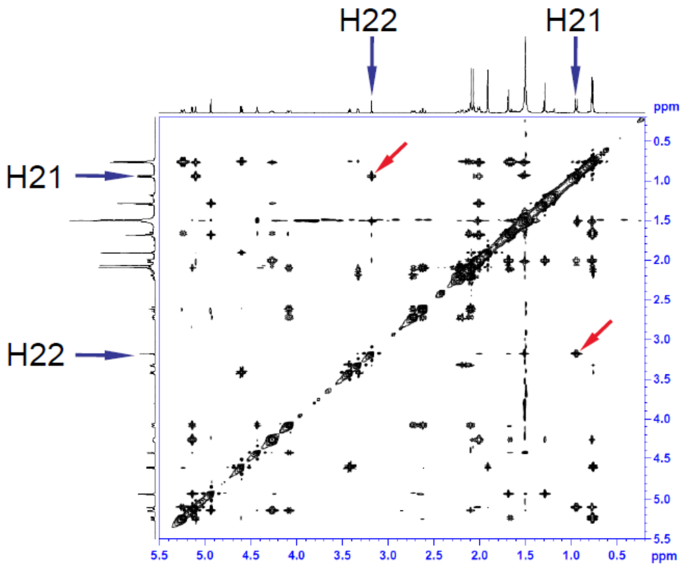

Supplementary Figure 4. **NOESY contour plot of taccalonolide AJ.** The NOE correlations between H21 and H22 are indicated by red arrows.

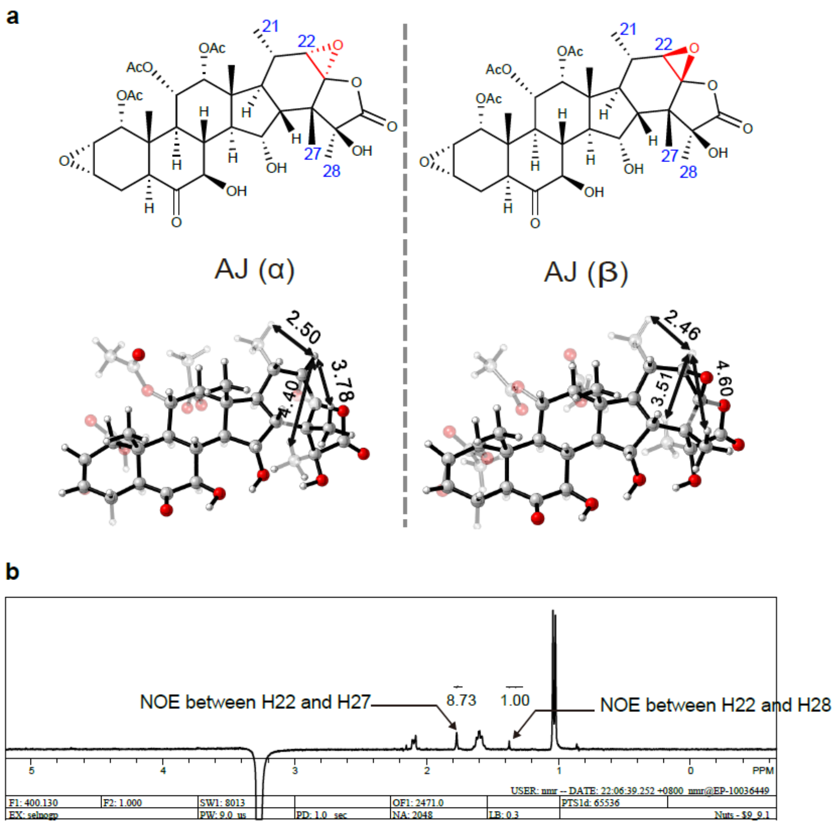

Supplementary Figure 5. The epoxide of taccalonolide AJ is in α configuration. (a) Gaussian calculations for taccalonolide AJ(α) and AJ(β). (b) Nuclear Overhauser Effect (NOE) of taccalonolide AJ , selectively labeled NOE correlations between H22 and H27 or H28.
